# Concussion in the National Hockey League: a systematic review of the literature

**DOI:** 10.2217/cnc.15.1

**Published:** 2015-08-06

**Authors:** Andrew W Kuhn, Gary S Solomon

**Affiliations:** 1 MedSport – Sports Medicine and Physical Therapy, University of Michigan Health System, 24 Frank Lloyd Wright Drive, Ann Arbor, MI 48106, USA; 2 Departments of Neurological Surgery, Orthopaedic Surgery & Rehabilitation, & Psychiatry, Vanderbilt Sports Concussion Center, Vanderbilt University School of Medicine, 1500 21st Avenue South, Neurosurgery Clinic, Suite 1506, Nashville, TN 37232, USA

**Keywords:** concussion, fighting, incidence, mechanism, National Hockey League

## Abstract

Players in the National Hockey League (NHL) are often sidelined by injuries, including concussion. The acute, intermediate and long-term effects of repetitive head trauma remain a concern of many. In 1997, the NHL and NHL Players Association established the NHL-NHL Players Association Concussion Program to diagnose, assess and treat concussion via a standardized and scientific approach. Documenting and analyzing the trends, incidence and underlying mechanisms of concussion may help in devising future prevention and treatment plans for concussion in hockey in general and the NHL in particular. The purpose of this study, therefore, was to systematically review and summarize the existing published literature on the trends, incidence rates and underlying mechanisms of concussion in the NHL.

Ice hockey is a fast, collision sport and players at the professional level are most often sidelined or slowed down by muscle sprains, ligamentous sprains, contusions, fractures and dislocations [1]. In a single 82-game National Hockey League (NHL) season, around 51% of all players miss at least one game due to significant injury [2]. Like most contact sports, concussion contributes significantly to injury rates and time loss from play in the NHL [2]. Concussion has been defined as a ‘complex pathophysiological process affecting the brain, induced by biomechanical forces’ [3]. Clinical signs and symptoms of concussion encompass physical, cognitive and emotional domains. They include signs such as gait disturbance, confusion and vomiting and symptoms such as headache, dizziness, difficulty remembering, irritability and unusual sleep patterns [4].

The possibility of long-term effects, resulting from multiple concussions, has been questioned and proposed in case reports and in case series of deceased athletes [5–9]. In a case series published in 2012, five former hockey players were found to have neuropathology deemed consistent with chronic traumatic encephalopathy (CTE) [9]. Although the most recent iteration of the international guidelines from the Concussion in Sport Group [3] concluded that “…a cause and effect relationship has not yet been demonstrated between CTE and concussions or exposure to contact sports,” the possibility of such a relationship still remains a cause of concern for many.

In 1997, the NHL and NHL Players Association (NHLPA) established the NHL-NHLPA Concussion Program to diagnose, assess and treat concussion via a standardized and scientific approach. Initially designed as a 5-year clinical research project, the program was incorporated into the general medical care of NHL players upon its completion and continues in existence to this date. Recently, experts in the field have gathered at the ‘Ice Hockey Summit: Action on Concussion’ to propose evidence based approaches in reducing the number of concussions in hockey [10]. Documenting and analyzing the trends, prevalence and underlying mechanisms of concussion may help in devising future prevention and treatment plans for concussion in hockey in general and the NHL in particular. The purpose of this study, therefore, was to systematically review and summarize the existing published literature on the trends, incidence rates and underlying mechanisms of concussion in the NHL.

## Methods

MEDLINE/PubMed, EMBASE and Cochrane Library records were searched to identify all studies related to concussion in the NHL. The following phrases were used as Medical Subject Heading and search terms for all databases: ‘professional hockey [AND] concussion’ and ‘national hockey league [AND] concussion’. The returned articles underwent a preliminary screening by the senior author. *A priori*, inclusionary criteria was defined as high-level evidence (Level I, II and III) empirical studies. Level IV (cross-sectional and case series) studies were included as well due to the limited published literature on concussion in the NHL. Exclusionary criteria included Level V evidence (case report and expert opinion) studies. Reference lists of the retrieved articles were examined for other pertinent sources.

## Results

### Literature search results

Eighty-two studies were returned from preliminary database searches. All studies were screened and nine met the specified inclusionary and exclusionary criteria [11–19]. Studies extracted, their experimental designs and corresponding levels of evidence are listed in Table 1.

**Table 1.** **Studies included for the systematic review.**

**Study**	**Study design**	**Level of evidence**	**Ref.**
Benson *et al.* (2011)	Prospective case series	IV	[11]

Donaldson *et al.* (2013)	Retrospective cross-sectional	IV	[12]

Hutchison *et al.* (2013)	Retrospective case series	IV	[13]

Hutchison *et al.* (2013)	Retrospective case series	IV	[14]

Pasternac *et al.* (2011)	Prospective cohort	III	[15]

Stevens *et al.* (2006)	Retrospective cohort	III	[16]

Stevens *et al.* (2008)	Retrospective cohort	III	[17]

Wennberg & Tator (2003)	Retrospective cross-sectional	IV	[18]

Wennberg & Tator (2008)	Retrospective cross-sectional	IV	[19]

### Incidence rate of concussion in the NHL (1986–2012)

**Figure 1.**
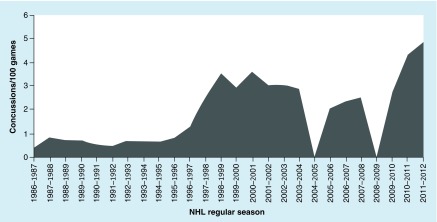
**Incidence rate of concussion in the National Hockey League: 1986–1987 through the 2011–2012 seasons.** The 2004–2005 season was locked out and thus no games were played. No data were reported for the 2008–2009 season. NHL: National Hockey League.

Four studies [11–12,18–19] reported on concussion incidence rates for regular season games in the NHL. With the exception of the 2004–2005 (lock-out) and 2008–2009 seasons, published incidence data were found for all other seasons between 1986 and 2012. The lowest incidence rate reported was during the 1986–1987 season (0.417 concussions/100 games). The second highest and highest were in 2010–2011 (4.350 concussions/100 games) and in 2011–2012 (4.878 concussions/100 games), respectively. Before the NHL lockout, the concussion incidence rate was 2.866 concussions/100 games. After the lockout, the rate dropped 28% to 2.073 concussions/100 games, but has since been rising. Rule 48, designed to eliminate checks to the head, was implemented at the beginning of the 2010–2011 season. However, the incidence rate of concussion steadily rose from 2009–2010 to 2011–2012. The data are tabularized in Table 2 and illustrated in Figure 1.

**Table 2.** **Incidence rate of concussion in regular season National Hockey League games: 1986–2012.**

**Study**	**Season**	**Concussions/100 NHL Games (SD)**	**Ref.**
Wennberg & Tator (2003)	1986–1987	0.417	[18]
	1987–1988	0.833	
	1988–1989	0.714	
	1989–1990	0.714	
	1990–1991	0.536	
	1991–1992	0.455	
	1992–1993	0.694	
	1993–1994	0.687	
	1994–1995	0.641	
	1995–1996	0.797	
	1996–1997	1.266	

Benson *et al.* (2011), Wennberg & Tator (2003), Wennberg & Tator (2008)	1997–1998	2.517 (0.456)	[11,18–19]
	1998–1999	3.538 (0.479)	
	1999–1900	2.947 (0.230)	
	2000–2001	3.618 (0.733)	
	2001–2002	3.049 (0.746)	

Benson *et al.* (2011), Wennberg & Tator (2008)	2002–2003	3.069 (0.201)	[11,19]
	2003–2004	2.886 (0.057)	
	2004–2005	NHL season lockout	

Wennberg & Tator (2008)	2005–2006	2.073	[19]
	2006–2007	2.358	
	2007–2008	2.520	
	2008–2009	No data reported	

Donaldson *et al.* (2013)^†^	2009–2010	2.764	[12]
	2010–2011	4.350	
	2011–2012	4.878	

^†^Includes ‘suspected concussions’ as determined by the authors.

NHL: National Hockey League; SD: Standard deviation.

### Incidence rate of concussion by position in the NHL

Three studies [11,13,19] reported on the incidence rate of concussion by position in the NHL. When the data were aggregated, forwards, defensemen and goalies comprised 63.3, 33.3 and 3.38% of concussions respectively. Data are presented in Table 3. In general, defensemen were most often concussed in their own defensive zone when retrieving the puck, whereas forwards sustained more concussions when ‘on the rush’ (carrying the puck into the offensive zone), ‘forechecking’ (pressuring opponents in the offensive zone) or ‘breaking out’ (carrying the puck out) of their own defensive zone [13].

**Table 3.** **Incidence rate of concussion by position in regular season National Hockey League games.**

**Position**	**Studies, n (%)**			**Pooled, n (%)**

	**Benson *et al.* (2011) [11]**	**Hutchison *et al.* (2013) [13]**	**Wennberg & Tator (2008) [19]**	
Forwards	341 (63.9)	129 (65.5)	428 (62.2)	898 (63.3)

Defensemen	169 (31.6)	63 (32.0)	241 (35.0)	473 (33.3)

Goalies	24 (4.49)	5 (2.54)	19 (2.76)	48 (3.38)

Total (n)	534	197	688	1419

NHL: National Hockey League.

### Incidence rate of concussion by mechanism in the NHL

Two studies [12,14] provided mode of mechanism by which concussions occur in the NHL. During the 2006–2010 seasons, body checking with head contact made up 62.1% of concussions. In the 2009–2012 seasons, after the implementation of Rule 48, body checking without head contact (31.7%) made up the greatest proportion of concussions sustained in the NHL. A more detailed analysis noted that concussed athletes had possession of the puck 23% of the time, did not have possession of the puck 34% of the time and 42% of the time the player had just released the puck. In over 70% of the cases, the time from puck release to contact was less than 0.5 s [14]. Another study found that 47% of concussions were ‘open ice’ (interior portion of the ice surface) events, 53% occurred around the ‘perimeter’ of the ice surface (including side dasher boards, corners, end boards and the side of the net) and 37% involved injured players’ heads contacting the dasher boards or plexiglass [13]. Unintentional and inadvertent mechanisms, such as getting hit by the puck or by an opponent’s stick, made up a small proportion of concussions. Fighting was responsible for around 9% of all concussions; however one study reported that the risk of concussion from fighting was 0.39% per fight [15]. The findings are summarized in Table 4.

**Table 4.** **Incidence rate of concussion by mechanism in the National Hockey League.**

**Mechanism of concussion**	**Studies, n (%)**	

	**Hutchison *et al.* (2013) [14]**	**Donaldson *et al.* (2013) [12]**
Body checking without head contact	50 (28.7)	39 (31.7)

Body checking with head contact	108 (62.1)	35 (28.5)

Blindsighting	N/A	5 (4.07)

Body checking with unknown head contact	N/A	5 (4.07)

Hit by opponents stick or skate	N/A	1 (0.81)

Hit By puck)	N/A	15 (12.2)

Unintentional	N/A	6 (4.88)

Unknown	N/A	6 (4.88)

Fighting	16 (9.20)	11 (8.94)

Total (n)	174	123

N/A: Not applicable.

### Equipment & other considerations for concussion in the NHL

In an analysis of 787 skating players (no goaltenders) during the 2001–2002 NHL season, there was no significant difference between the number of concussions sustained by players who wore visors and player who did not wear visors [16]. Similarly, another study found that about half of the players who received concussions were wearing a visor [13]. In 2008, Stevens *et al.* [17] found that when examining NHL players from the 2001 to 2002 season, total ice time per game was a significant predictor of concussion, while total ice time per season was not.

## Discussion

The purpose of this systematic review was to summarize the limited literature regarding the incidence rates, trends and underlying mechanisms of concussion in the NHL. Furthermore, this review serves to provide an evidence base for future concussion prevention and treatment programs in the NHL.

The rate of concussion in the NHL steadily rose greater than tenfold from 1986–1987 to 2011–2012. One possible explanation for this trend is that players are becoming stronger, taller and heavier, contributing to this increased rate. From 1986–1987 to 2001–2002, mean NHL player height increased by one inch and average weight increased by 10 pounds [18]. However, the current average height and weight of active NHL players is 6’1” and 202.6 pounds [20], which has remained relatively unchanged when compared with published data for the 1997–1998 through the 2001–2002 seasons [18]. While players may have simply become more violent and tactical in the way they body check opposing players, another possible and more likely, explanation may lie in how the definition, diagnosis, reporting biases and management of concussion have evolved over the past three decades. Given recent cases reporting neurophysiological abnormalities of brain tissue in decreased athletes [5–7,21], in addition to documentation of subjective [22–24] and objective [25–28] effects potentially due to repetitive head trauma, athletes and physicians may have become more alert, cautious and vigilant in reporting and managing concussion. Indeed, the definition of concussion used by the NHL Concussion working group has evolved from the initial 1997 American Academy of Neurology definition (which essentially involved traumatically induced mental status changes with or without loss of consciousness) to the multidimensional definitional iterations proffered by the Concussion in Sport Group [3].

Rule 48 was implemented prior to the 2010–2011 season and aimed to eliminate hits to the head as a preventative measure for concussion. While fewer concussions resulted from high head hits, the total number of concussions continued to increase. A particularly noteworthy finding from the current review was when most concussions occur. In the majority of cases, it was found that the concussed player had just released the puck with the hit coming less than half a second later. Concussion may result more often when the player is unaware, unexpecting and unable to prepare for an oncoming check. Fighting was found to contribute only a small amount to the number of concussions and of these, 75% included secondary head contact (i.e., head hitting the ice) [14].

At any one time during even-strength play, there are three forwards, two defensemen and one goalie per team on the ice. It is expected that the proportion of concussions would be roughly 50% for forwards, 33.3% for defensemen and 16.7% for goalies. NHL forwards and goalies however seem to sustain greater and lesser proportions of concussions than expected, respectively. Forwards are required to play defensively in their own zone, breakout through the neutral zone and score goals in the offensive zone. These results may be explained by the hypothesis that forwards cover and skate over more ice than goalies or defensemen, subjecting themselves to a higher probability of collision and concussion. While visors may provide benefit in preventing orbital or facial injuries, the data to date indicates that their utility in preventing concussion appears limited. Lastly, fatigue may be a factor in concussion incidence as well. In one study covering one season, time on ice per game was a significant predictor of concussion, whereas time on ice per season was not. Ensuring that players are conditioned and ready for the physical demands of a NHL regular season game may be beneficial in preventing concussion.

As this study was a systematic review of the rare literature reporting on concussion in the NHL, limitations exist. As mentioned, there are not many high level evidence-based empirical studies on concussion in the NHL because of a small elite population and limited available data. We therefore included retrospective and prospective case series reports in this review. Second, how these studies defined and diagnosed concussion varied methodologically. Hutchison *et al.* [29] developed and utilized a ‘heads-up checklist’ methodology for the retrospective analysis of concussions via video. Others used all possible sources of information such as the team’s published report, or secondary media sources like Rotoworld, the Sports Network, Yahoo Sports, CBS Sports, local newspapers and official team websites [12]. When analyzing mechanisms of concussion, differences also existed in methodology and classification. Lastly, this study focused exclusively on NHL players, and the results may not be generalizable to other populations.

One area for empirical consideration and future study of preventative measures for concussive injury may lie in adapting the rink size of NHL play. Rinks in the NHL measure 200 feet long and 85 feet wide. International sized rinks (utilized in international tournaments, the Olympics and European leagues) also measure 200 feet long, but are 100 feet wide, which equates to an extra 3000 square feet of ice on which to play. In 2004, Wennberg [30] examined games from the Stanley Cup Finals (NHL), the Winter Olympics and World Junior Championship. He found that there were significantly less total collisions, player/player body checks, player/player collisions into the boards, player/player open ice collisions, total head collisions, indirect head force, direct head impact from player or boards or stick and puck and severe head impacts in games played on international sized rinks when compared with games played on the NHL sized rink. In another study [31], it was found that when adjusting for sample size, North American players in the NHL accumulated significantly more penalty minutes than European players. North American players most likely grew up playing on NHL sized ice, whereas Europeans probably grew up playing on international sized rinks. It is possible that different playing styles developed, at least in part, as a function of rink size. Physicality and toughness may be more developed in North American players because of decreased ice surface and closer proximity to other players, whereas speed and passing may be more prevalent in European players due to the increased width of ice. Additionally, since many concussions occur after a player releases the puck, rule changes designed to protect the puck carrier may lead to a decrease in concussions.

## Conclusion & future perspective

Systematic reviews generally retain the evidence level of the lowest level paper(s) reviewed. In effect, the current study is a Level IV systematic review. While the trend of increasing concussions may be due to heightened awareness, diagnosis, reporting biases and management, these findings lend credence to the belief that reducing the number of concussions may not be achieved alone by penalizing hits to the head or eliminating fighting from the game. Reducing the number of concussions in the NHL must be achieved in different ways and deciding on preventative measures should be an evidence-based approach [10].

Physicality is a part of hockey and always will be. By no means are the authors suggesting that the physical nature of hockey be eliminated completely. However, the increased prevalence of concussion appears to have not been tamed by rule changes and ongoing debate over fighting. There were no level I or II studies in the literature to review or from which to base recommendations. While future systematic prospective study is needed, the current literature review raises the possibilities that increasing rink size in the NHL and offering greater protection to the puck carrier may be viable options in reducing the number of concussions in the NHL, and conceivably at the youth, high school and collegiate levels in North America.

**Executive summary**

**Background**

- Concussion is prevalent in professional sporting leagues like the National Hockey League (NHL).
- The possibility of intermediate- and long-term effects from repetitive head trauma remain a concern of many.
- In 1997, the NHL and NHL Players Association (NHLPA) established the NHL-NHLPA Concussion Program to diagnose, assess and treat concussion via a standardized and scientific approach.
- Documenting and analyzing the trends, prevalence and underlying mechanisms of concussion may help in devising future prevention and treatment plans for concussion in hockey in general and the NHL in particular.

**Incidence**

- The rate of concussion in the NHL steadily rose greater than tenfold from 1986–1987 to 2011–2012.

**Trends**

- Forwards, defensemen and goalies comprised 63.3, 33.3 and 3.38% of concussions, respectively.
- Visor usage provides limited utility in preventing concussion.
- Conditioning may be a pertinent factor in concussion.

**Mechanisms**

- Rule 48 was implemented at the beginning of the 2010–2011 season to halt the number of direct hits to the head. While direct head hits contributed to concussions less frequently, the incidence rate of concussion steadily rose from 2009–2010 to 2011–2012.
- Fighting accounted for about 9% of concussions in the NHL. Seventy-five percent of these were from secondary head contact, such as a player’s head striking the ice when falling.
- Most concussions occurred around the perimeter, from players’ heads hitting the glass or boards, and just after the puck is released.

**Conclusion & future perspective**

- The trend of increasing concussions may be due to heightened awareness, diagnosis, reporting biases and concussion management
- These findings also lend credence to the hypothesis that reducing the number of concussions may not be achieved alone by penalizing hits to the head or eliminating fighting from the game.
- Reducing the number of concussions in the NHL must be achieved in different ways and deciding on preventative measures should be an evidence-based approach
- While further study is needed, this review suggests that the variables of rink size and offering greater protection to the puck carrier may be viable options in reducing the number of concussions in the NHL, and conceivably at the youth, high school and collegiate levels in North America.
- It will require a collaborative effort between the NHL and the NHLPA to consider the scientific evidence base in considering rule modifications to decrease the frequency of concussive injury.
